# Change of Apoptosis and Glucose Metabolism in Lung Cancer Xenografts during Cytotoxic and Anti-Angiogenic Therapy Assessed by Annexin V Based Optical Imaging and ^18^F-FDG-PET/CT

**DOI:** 10.1155/2021/6676337

**Published:** 2021-04-10

**Authors:** Jasmin Gross, Karin Palmowski, Dennis Doleschel, Anne Rix, Felix Gremse, Frederic Verburg, Felix M. Mottaghy, Fabian Kiessling, Wiltrud Lederle, Moritz Palmowski

**Affiliations:** ^1^Institute for Experimental Molecular Imaging, RWTH Aachen University, Aachen, Germany; ^2^Department of Nuclear Medicine, RWTH Aachen University, Aachen, Germany; ^3^Department of Radiology and Nuclear Medicine, Maastricht University Medical Center (MUMC+), Maastricht, Netherlands

## Abstract

**Methods:**

For apoptosis imaging, the near-infrared probe Annexin Vivo750 was used in combination with fluorescence molecular tomography and microcomputed tomography (FMT/*µ*CT). Glucose metabolism was assessed using ^18^F-FDG-PET/CT. Five groups of nude mice bearing lung cancer xenografts (A549) were investigated: (i) untreated controls and two groups after (ii) cytotoxic (carboplatin) or (iii) anti-angiogenic (sunitinib) treatment for four and nine days, respectively. Imaging data were validated by immunohistochemistry.

**Results:**

In response to carboplatin treatment, an inverse relation was found between the change in glucose metabolism and apoptosis in A549 tumors. Annexin Vivo showed a continually increasing tumor accumulation, while the tumor-to-muscle ratio of ^18^F-FDG continuously decreased during therapy. Immunohistochemistry revealed a significantly higher tumor apoptosis (*p*=0.007) and a minor but not significant reduction in vessel density only at day 9 of carboplatin therapy. Interestingly, during anti-angiogenic treatment there was an early drop in the tumor-to-muscle ratio between days 0 and 4, followed by a subsequent minor decrease (^18^F-FDG tumor-to-muscle-ratio: 1.9 ± 0.4; day 4: 1.1 ± 0.2; day 9: 1.0 ± 0.2; *p*=0.021 and *p*=0.001, respectively). The accumulation of Annexin Vivo continuously increased over time (Annexin Vivo: untreated: 53.7 ± 36.4 nM; day 4: 87.2 ± 53.4 nM; day 9: 115.1 ± 103.7 nM) but failed to display the very prominent early induction of tumor apoptosis that was found by histology already at day 4 (TUNEL: *p*=0.0036) together with a decline in vessel density (CD31: *p*=0.004), followed by no significant changes thereafter.

**Conclusion:**

Both molecular imaging approaches enable visualizing the effects of cytotoxic and anti-angiogenic therapy in A549 tumors. However, the early and strong tumor apoptosis induced by the anti-angiogenic agent sunitinib was more sensitively and reliably captured by monitoring of the glucose metabolism as compared to Annexin V-based apoptosis imaging.

## 1. Introduction

For personalized cancer therapy, an early discrimination between responders and non-responders is of high importance to tailor the treatment and improve the therapeutic benefit [1]. The standard methodology to evaluate treatment response is based on anatomic imaging of the tumor size [2, 3]. However, changes in tumor size usually occur with delay to the treatment, and thus, tumor size measurements are not well suited for early therapy assessment. Hence, there is a need for alternative noninvasive imaging approaches capable of reliably indicating early signs of treatment response.

For the assessment of early therapy effects, molecular imaging of the glucose metabolism using ^18^F-FDG-PET/CT is superior with respect to standard assessment of tumor volume reduction [3, 4], since metabolic changes usually occur earlier in response to therapies. In many clinical studies, ^18^F-FDG-PET/CT has been shown to predict tumor response, progression-free survival, and overall survival with high accuracy [1, 5]. However, the glucose uptake is influenced by various parameters, e.g., by the size of the tumors or the blood glucose levels, and these parameters can affect the reliability of ^18^F-FDG-PET imaging [6, 7]. In addition, for tumors with a low basal glucose metabolism, assessment of therapy effects by ^18^F-FDG-PET imaging is very difficult [8]. Additionally, an increase in the ^18^F-FDG uptake is observed in tumors in response to therapies that induce a prominent immune reaction; thus a positive response to the therapy is not always associated with a decrease in the ^18^F-FDG uptake [7, 9].

As an alternative approach, molecular imaging of tumor apoptosis can be applied to assess anti-cancer drug response. Most commonly, apoptosis imaging is based on targeting exposed phosphatidylserines by derivatives of Annexin V and different Annexin V-based imaging probes and tracers have been developed and used in preclinical studies and even in clinical trials [10, 11]. Annexin V is a protein with a molecular weight of 35.8 kDa that binds to phosphatidylserines with high affinity. In healthy cells, phosphatidylserines are mostly localized in the inner leaflet of the cell membrane. But within the first few hours after apoptosis induction, phosphatidylserines are redistributed to the outer leaflet of the cell membrane and thereby become accessible to Annexin V and Annexin V-based imaging probes [12]. Annexin V-based apoptosis imaging has been shown to reliably detect therapy-induced tumor apoptosis in various preclinical studies [13–16] and was extensively evaluated in patients [17–20].

However, in our previous study in a skin cancer xenograft model with a highly angiogenic and immature vasculature (A431), we have shown that the delivery of Annexin V-based imaging probes can be impeded by strong anti-angiogenic therapy effects, thus hampering the reliability of this molecular imaging approach. Here, the strong reduction of the tumor vessels induced by the anti-angiogenic drug sunitinib resulted in a low intratumoral accumulation of the Annexin V-based imaging probe despite strong induction of apoptosis [21]. To further understand the pitfalls of Annexin V-based apoptosis imaging, we were highly interested in exploring, whether these findings can be reproduced in a less angiogenic tumor model with a more mature vasculature that is less responsive to anti-angiogenic drugs and therefore more reliably reflect tumors in patients.

Thus, in this study, we used the lung cancer xenograft model A549 which is less angiogenic than the A431 model (vascularization in A431 tumors is about 3x higher than in A549) and has a remarkably higher amount of mature vessels (>70% in A549 vs. 14% in A431) [22]. The tumors were treated either with the cytostatic drug carboplatin, targeting proliferating cells, or the strong anti-angiogenic multi-tyrosine kinase inhibitor sunitinib. Annexin V-based apoptosis optical imaging was performed before and at days 4 and 9 after therapy, and results were compared to changes in glucose metabolism assessed by ^18^F-FDG-PET/CT.

## 2. Methods

### 2.1. Tumor Inoculation and Therapy

The animal experiments were performed according to German legal requirements and animal protection laws and were approved by the Governmental Review Committee on Animal Care. Mice received a standard diet and were housed in individually ventilated cages under specific pathogen-free conditions in a humidity- and temperature-controlled environment in groups of 3 to 5 animals per cage with a light and dark cycle of 12 h, respectively, according to the guidelines of “Federation for Laboratory Animal Science Associations.”

**Figure 1:**
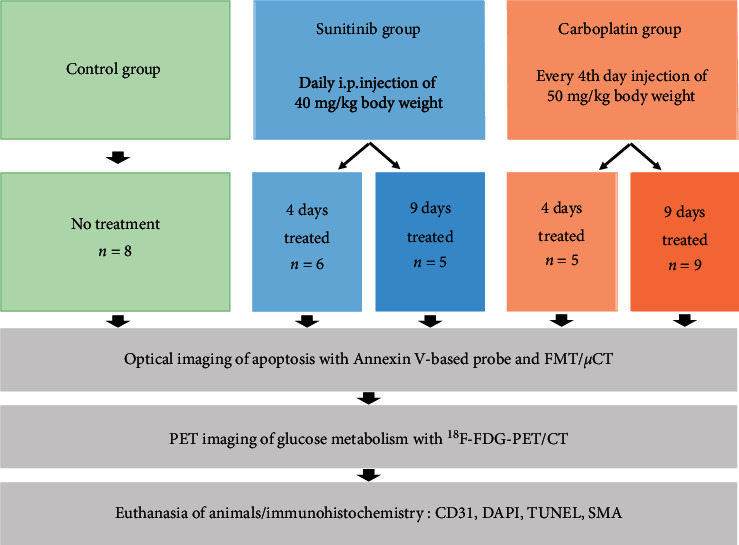
Overview of the study design. Five groups of lung cancer xenograft (A549) bearing mice were investigated: (i) untreated controls and treated groups after (ii) anti-angiogenic (SU11248) or (iii) cytotoxic (carboplatin) treatment for four and nine days. Optical imaging of apoptosis using an Annexin V-based probe was performed with FMT/*µ*CT, followed by ^18^F-FDG-PET/CT. Imaging data were validated by quantitative immunohistochemistry.

Human non-small-cell lung carcinoma xenografts were induced by s.c. injection of 4 × 10^6^ A549 cells in the right limb of female CD1 nude mice (age 6–8 weeks; Charles River). When the tumors had reached a mean size of 60 mm^3^, animals were divided randomly into five groups. The control group (*n* = 8) remained untreated and was measured directly by noninvasive imaging (d0) [23]. For anti-angiogenic treatment, the mice received daily intraperitoneal injections (i.p.) of 40 mg/kg body weight of the clinically approved multispecific tyrosine kinase inhibitor sunitinib (SU11248, Pfizer, Inc.; dissolved in 60 *μ*L DMSO and 30 *μ*L PBS) as described [24]. Animals of the chemotherapy group were treated by i.p. injections of carboplatin (50 mg/kg body weight) every 4^th^ day. Treatment was performed for 4 days (*n* = 6 for sunitinib; *n* = 5 for carboplatin) and for 9 days (*n* = 5 for sunitinib; *n* = 9 for carboplatin). Imaging was performed at days 4 and 9. The study design is summarized in Figure 1.

### 2.2. Apoptosis Imaging by Combined Fluorescence Molecular Tomography and Microcomputed Tomography (FMT/*µ*CT)

Fluorescence molecular tomography (FMT) and microcomputed tomography (*µ*CT) scans were performed *in vivo*, 2 hours after i.v. injection of 2 nmol of the commercially available Annexin V-based imaging probe Annexin Vivo 750 (PerkinElmer). Mice were scanned and transported between the devices using a multi-modality mouse bed while being anesthetized with isoflurane during the whole procedure [23]. For better tumor localization, a *µ*CT scan was performed directly before the FMT measurement. Both tubes of the dual energy *µ*CT (Tomoscope Duo, CT Imaging GmbH) were run at energies of 65 kV and currents of 0.38 mA. Each flat panel detector acquired 720 projections at 25 frames per second during a full rotation with a total scan time of 29 seconds. A Feldkamp algorithm was used for image reconstruction with isotropic voxel sizes of 70 *µ*m and a smooth reconstruction kernel. FMT (FMT 2500, Perkin Elmer) was performed using a wave-type specific scanner for transillumination, reflectance, and absorption as described [25]. Tomographic data sets of *µ*CT and FMT were fused during the post-processing step using fiducial markers in the multi-modality mouse bed [23]. The tumor was segmented in 3D based on the anatomical *µ*CT-data to quantify the amount of co-localized fluorescence (Imalytics Preclinical, Gremse-IT GmbH) [26].

### 2.3. Measurement of Glucose Metabolism by ^18^F-FDG-PET/CT

PET/CT acquisition was performed at a Philips Gemini TF 16 PET/CT system (Philips Medical Systems) [27]. The system includes a time-of-flight (TOF) capable, fully three-dimensional (3D) PET scanner with a transaxial and axial full width half maximum (FWHM) of 4.3 mm (incorporating the effects of Line of Response (LOR) reconstruction) combined with a 16-slice CT scanner. Data sets were reconstructed using the iterative LOR-TF-RAMLA/3i/33s (“Blob-OS-TF”) algorithm, resulting in 90 images with 128 × 128 voxels of 2 × 2 × 2 mm^3^. Data sets were fully corrected for random coincidences, scatter radiation, and attenuation. For data analysis, PET images were fused with the CT-data (120 kV, 80 mA, slice thickness 1 mm).

Animals of all groups were prepared for injection of ^18^F-FDG according to the recommendations of Fueger et al. [28]. They were anesthetized by 2% isoflurane inhalation and the animal cage was warmed up. ^18^F-FDG (7 MBq in 0.2 mL saline solution) was injected i.p. under anesthesia. Warming and general anesthesia continued throughout the entire uptake and examination period. 1 h after the injection, the animals were placed in a special mouse bed which was transferred in the PET/CT scanner, allowing analyses of four animals per scan as described [27]. Based on the PET/CT images, the tracer uptake was measured in tumor and muscle and the tumor-to-muscle ratio was determined.

### 2.4. Immunohistochemical Analyses

Animals were euthanized after imaging. Tumors were resected, frozen in liquid nitrogen vapor, and cut in slices of 8–10 *μ*m. Fixation of the frozen sections and the staining procedure were performed as described [29]. For staining, primary antibodies against CD31 (rat anti-mouse PECAM-1; BD Biosciences), SMA (biotinylated mouse anti-SMA, Progen), and collagen IV (rabbit anti-collagen IV, Novotec) as well as corresponding secondary antibodies were used [27]. Cell nuclei were counterstained by 4′,6-diamidino-2-phenylindole (DAPI; Invitrogen). Apoptotic cells in the tissue were detected by TUNEL staining using the “In Situ Cell Death Detection Kit, TMR red” (Roche Diagnostics). Stained sections were examined and photographed (Zeiss Axio Imager M2, Carl Zeiss, MicroImaging GmbH).

### 2.5. Morphometric Analysis

Blood vessel density in s.c. tumors was quantified by determining the ratio of the CD31+ area fraction to the total area, using fifteen sections at 20x magnification per slice for covering the whole tumor area. The apoptotic cell rate was determined by dividing the TUNEL-positive area fraction by the total DAPI positive area, using five sections per tumor slice at 10x magnification. Mature vessels were quantified by counting CD31 and double CD31 and SMA-positive vessels and determining the ratio of SMA-positive vessels using five sections per tumor slice at 20x magnification.

### 2.6. Statistical Evaluation

Data are presented as mean ± standard deviation. Statistical analysis was done using a Kruskal-Wallis test and a Dunn's post-hoc test for multiple comparisons. *P* values of <0.05 and <0.01 were considered as significant (^∗^) and highly significant (^∗∗^) differences, respectively. Statistical analysis was done with GraphPad Prism (GraphPad Software).

## 3. Results

### 3.1. Tumor Accumulation of Annexin Vivo and Tumor-to-Muscle Ratio of ^18^F-FDG in Response to Carboplatin Treatment

**Figure 2:**
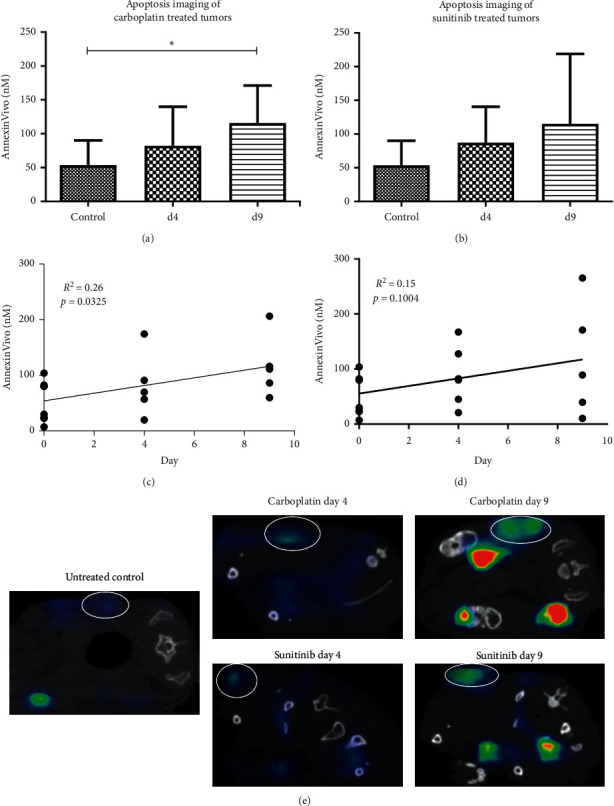
Molecular imaging of apoptosis. (a) Quantitative analysis demonstrates a significantly higher accumulation of AnnexinVivo at day 9 after therapy start with carboplatin (*p*=0.011). (b) In sunitinib-treated tumors, AnnexinVivo accumulation also increases continuously; however, differences to the controls are not significant. (c) Linear regression analysis of the accumulation of AnnexinVivo during carboplatin treatment showing a significant increase in the accumulation of AnnexinVivo over time (*R*^2^ = 0.26, slope: 6.91, *p*=0.0325). (d) Linear regression analysis of the accumulation of AnnexinVivo during sunitinib treatment. An increase in the accumulation of AnnexinVivo is visible over time, though not significant (*R*^2^ = 0.15, slope: 6.88, *p*=0.1004). (e) Representative FMT/*µ*CT hybrid images (transverse plane) showing the signal of AnnexinVivo in the tumor of an untreated control animal and in tumors of carboplatin and sunitinib-treated animals at day 4 and day 9. The accumulation of AnnexinVivo increases in the tumors in response to both drugs over time.

Annexin V-based apoptosis imaging was evaluated with respect to the assessment of early and prolonged therapy effects at days 4 and 9 during treatment with the chemotherapeutic drug carboplatin in A549 lung cancer xenografts. In parallel, changes in the glucose metabolism were monitored by PET/CT imaging using the clinically well-established tracer ^18^F-FDG. Untreated animals were included as controls and measured once before therapy start by noninvasive imaging. When assessing apoptosis *in vivo* using Annexin Vivo750 and FMT/*µ*CT hybrid imaging, a continuously increasing accumulation of Annexin Vivo was found in carboplatin-treated tumors during the observation period (control group: 53.69 ± 36.44 nM Annexin Vivo, d4: 82.29 ± 57.67 nM Annexin Vivo, d9: 140.1 ± 76.79 nM Annexin Vivo). Differences to the controls were significant at day 9 after therapy start (day 9: *p* = 0.011) (Figure 2(a)). Regression analysis of individual values showed a significant increase in the accumulation of Annexin Vivo in the tumors over time for carboplatin (*R*^2^ = 0.26, *p* = 0.0325, slope: 6.91 nM/day) (Figure 2(c)).

**Figure 3:**
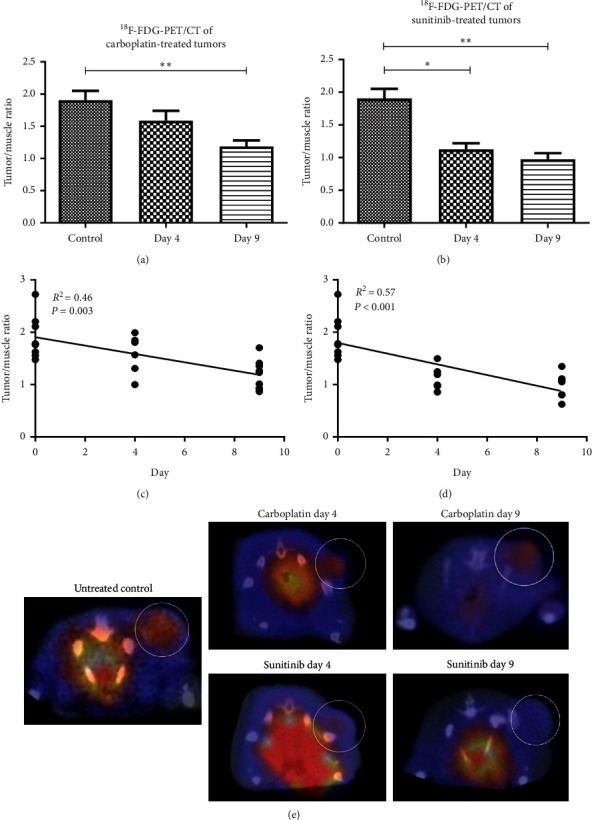
Measurement of the glucose metabolism. Quantitative analysis demonstrates decreasing tumor-to-muscle ratio of ^18^F-FDG during treatment with carboplatin and sunitinib, respectively. In sunitinib-treated tumors, differences to the controls were significant and highly significant at therapy days 4 and 9 (*p*=0.021 and *p*=0.001, respectively) (b), whereas in carboplatin-treated tumors, the tumor-to-muscle ratio of ^18^F-FDG uptake was significantly lower than in the control tumors at day 9 after therapy start (a). (c) Linear regression analysis showing a significant decrease in the tumor-to-muscle ratio of ^18^F-FDG during carboplatin treatment (*R*^2^ = 0.46, slope: −0.08, *p*=0.003). (d) A significant decrease in the tumor-to-muscle ratio of ^18^F-FDG is observed during sunitinib treatment in the linear regression analysis (*R*^2^ = 0.57, slope: −0.10, *p* < 0.001). (e) Representative ^18^F-FDG-PET-CT images (transverse plane) showing high ^18^F-FDG uptake in the tumor of an untreated control mouse and a decrease in the uptake in carboplatin and sunitinib-treated tumors over time.

Assessment of the glucose metabolism by ^18^F-FDG-PET/CT imaging revealed a continuous decrease in the tumor-to-muscle ratio in response to carboplatin treatment (tumor-to-muscle ratios: control group: 1.90 ± 0.41; day 4: 1.58 ± 0.37, d9: 1.18 ± 0.27) (Figure 3(a)). Similarly to the accumulation of Annexin Vivo, differences in the tumor-to-muscle ratio between treated and untreated animals were significant at day 9 after therapy start (*p*=0.0046). In the regression analysis, a significant decrease in the tumor-to-muscle ratio was obtained during carboplatin treatment (*R*^2^ = 0.46; *p* = 0.003, slope: −0.08/day) (Figure 3(c)).

### 3.2. Tumor Accumulation of Annexin Vivo and Tumor-to-Muscle Ratio of ^18^F-FDG in Response to Sunitinib Treatment

Next, we evaluated apoptosis imaging using Annexin Vivo750 and FMT/*µ*CT imaging in response to anti-angiogenic therapy with sunitinib. Again, the glucose metabolism was analyzed by ^18^F-FDG-PET/CT imaging. Comparable to the results obtained for cytotoxic therapy, the accumulation of Annexin Vivo in tumors continuously increased in response to sunitinib treatment (control group: 53.69 ± 36.44 nM Annexin Vivo; day 4: 87.20 ± 53.36 nM Annexin Vivo; day 9: 115.08 ± 103.69 nM Annexin Vivo) (Figure 2(b), representative FMT/*µ*CT hybrid images are shown in Figure 2(e)). Regression analysis showed an almost similar slope (6.876 nM/day) for the change over time as being observed in response to carboplatin (6.909 nM/day). Nevertheless, the increase in Annexin Vivo accumulation in tumors during sunitinib treatment was not significant (*R*^2^ = 0.15, *p*=0.1004) (Figure 2(d)).

Analysis of the glucose metabolism by ^18^F-FDG-PET/CT revealed a markedly and significantly lower tumor-to-muscle ratio in sunitinib-treated versus control mice already at day 4 after therapy start. A further minor decrease was observed between treatment days 4 and 9 (tumor-to-muscle ratios: untreated control group: 1.90 ± 0.41; day 4: 1.12 ± 0.23; day 9: 0.97 ± 0.24, *p* = 0.021 and *p* = 0.001, respectively) (Figure 3(b), representative PET/CT images are shown in Figure 3(e)). Regression analysis revealed a significant decrease in the tumor-to-muscle ratio over the treatment period (*R*^2^ = 0.57, *p* < 0.001, slope: −0.102/day) (Figure 3(d)). The decline in response to sunitinib was steeper as compared to carboplatin (slope sunitinib: −0.102/day; slope carboplatin: −0.080/day).

### 3.3. Immunohistochemistry: Tumor Apoptosis in Response to Sunitinib and Carboplatin Treatment

**Figure 4:**
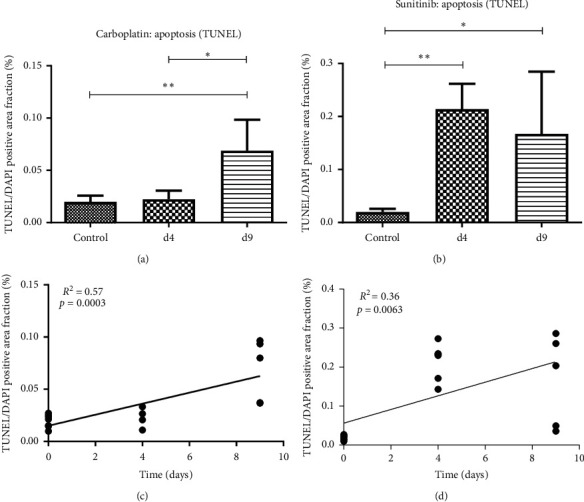
Quantitative immunohistochemistry. (a, b) TUNEL staining demonstrates the induction of tumor apoptosis in response to both drugs; however, sunitinib induces an earlier and stronger apoptosis in the tumors. For carboplatin-treated tumors, differences to the controls are significant only at day 9 (*p*=0.007), whereas apoptosis is already significantly increased in sunitinib-treated tumors at treatment day 4 (*p*=0.0036 for day 4, *p*=0.0137 for day 9). (c) Linear regression analysis showing a significant increase in the TUNEL/DAPI positive area fraction during carboplatin treatment (*R*^2^ = 0.57, slope: 0.01, *p*=0.0003). (d) A significant increase in the TUNEL/DAPI positive area fraction is observed during sunitinib treatment in the linear regression analysis (*R*^2^ = 0.36, slope: 0.02, *p*=0.0063).

The *in vivo* imaging data were validated by immunohistochemical analyses of the resected tumors. For carboplatin, apoptosis in the treated tumors was almost comparable to the controls at day 4 after therapy start (TUNEL + area fraction: carboplatin: control group: 0.02 ± 0.01, d4: 0.02 ± 0.01). Tumor apoptosis significantly increased between treatment days 4 and 9 (TUNEL + area fraction: carboplatin d9: 0.07 ± 0.03; d4 vs. d9: *p* = 0.007; control vs. d9: *p* = 0.033) (Figure 4(a)).

**Figure 5:**
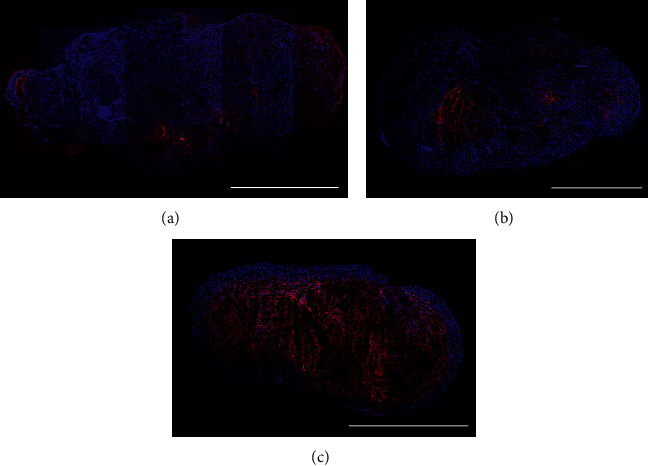
Representative TUNEL stainings of entire tumor sections. Images showing TUNEL stainings (TUNEL in red, counterstaining of nuclei by DAPI in blue) of entire sections from a representative tumor of the control group (a), the cytotoxic (carboplatin, (b)), and the anti-angiogenic (sunitinib, (c)) treatment group at day 4. In the carboplatin-treated tumor, apoptosis is only slightly higher than in the untreated control tumor. In the sunitinib-treated tumor, however, apoptosis is strongly enhanced. Bar: 1 mm.

In response to sunitinib treatment, a strong induction of apoptosis was observed in the tumors already at day 4 with a 10-fold higher mean value as compared to the controls (TUNEL + area fraction: control group: 0.02 ± 0.01; d4: 0.21 ± 0.04; *p* = 0.0036). Tumor apoptosis remained significantly enhanced at day 9 (d9: 0.16 ± 0.11, *p* = 0.0137) **(**Figure 4(b), representative TUNEL stainings of tumor sections are shown in Figure 5).

Regression analysis showed a significant increase in tumor apoptosis over time for both treatments (carboplatin: *R*^2^ = 0.566, *p* = 0.0003; sunitinib: *R*^2^ = 0.363, *p* = 0.006) (Figures 4(c) and 4(d)). The slope was higher for sunitinib (0.018%/day) as compared to carboplatin treatment (0.005%/day).

### 3.4. Immunohistochemistry: Treatment Effects of Sunitinib and Carboplatin on Tumor Blood Vessels

**Figure 6:**
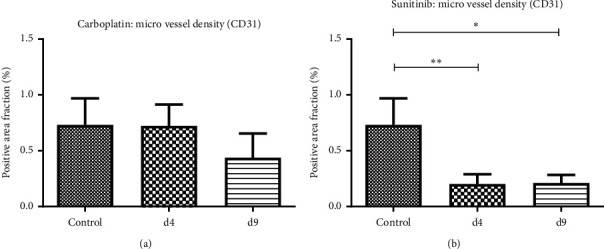
Micro vessel density in tumors. Quantitative analysis of the CD31+ area fraction confirms the strong anti-angiogenic effect of sunitinib (*p*=0.004 and *p*=0.011 at treatment days 4 and 9, respectively) (b) whereas carboplatin exerts minor effects on the tumor vasculature (a).

In order to assess the effects of both drugs on tumor vascularization, we quantified the blood vessel density in tumor sections (CD31 stained vessels). In response to sunitinib, a strong and highly significant reduction in vessel density was observed at treatment day 4 and the vessel density remained at a low level at day 9 (Figure 6(b): CD31+ area fractions: control group: 0.73 ± 0.23%; sunitinib-treated group d4: 0.20 ± 0.08%; *p* = 0.004 and d9: 0.21 ± 0.07%; *p* = 0.011). In carboplatin-treated tumors, vessel density was comparable to the control tumors at day 4 (d4: 0.72 ± 0.19%; *p* > 0.999). At day 9 of carboplatin treatment, blood vessel density was noticeably but not significantly reduced (d9: 0.43 ± 0.21%; *p* = 0.132) (Figure 6(a)). Thus, the decrease in blood vessel density in response to sunitinib treatment was stronger as compared to carboplatin and occurred earlier.

Further analysis revealed that sunitinib led to a noticeable but not significant reduction in the amount of mature vessels (SMA-positive vessel counts: controls: 55.57 ± 19.87; sunitinib d4: 41.33 ± 24.93; d9: 34.70 ± 21.25), whereas carboplatin treatment had no major effects (SMA-positive vessel counts: carboplatin d4: 61.48 ± 10.25; d9 54.38 ± 17.22) (Supplementary Figure S1).

## 4. Discussion

Annexin V-based apoptosis imaging has been proposed as a promising approach for the early discrimination between responders and non-responders to cancer therapy [30]. However, in our previous study in a highly angiogenic tumor model (A431), we have shown that the reliability of Annexin V-based apoptosis imaging for treatment response assessment can be compromised in case of strong anti-angiogenic therapy that significantly reduces tumor blood vessels, thus impairing the delivery of the imaging probe to the tumor despite strong induction of apoptosis. To gain further insight into the potential and limitations of Annexin V-based apoptosis, we evaluated this approach in response to anti-cancer drugs that exert differential effects on the tumor tissue: the chemotherapeutic agent carboplatin and the strong anti-angiogenic drug sunitinib. In addition, we used a less angiogenic tumor model (A549) with a higher degree of vessel maturity which is closer to human tumors in patients. Annexin V-based tumor apoptosis imaging was compared with glucose metabolism imaging by ^18^F-FDG-PET/CT which is used in the clinics.

Both imaging approaches, the assessment of the glucose metabolism and Annexin V-based apoptosis imaging, enabled monitoring treatment effects in response to cytotoxic therapy. A continuous increase in tumor accumulation of Annexin Vivo during carboplatin treatment coincided with a continuous decrease in tumor-to-muscle ratio of ^18^F-FDG. For both imaging approaches, differences in probe uptake between treated and untreated tumors were significant at day 9, which is in agreement with the immunohistochemical data. Our findings are in line with results obtained in a hepatoma rat model [31] and a clinical breast cancer study [32]. In both studies, a decrease in ^18^F-FDG tumor uptake negatively correlated with an increase in radioactively labelled ^99m^Tc-Annexin V levels during cytotoxic chemotherapy.

For anti-angiogenic therapy with sunitinib, both imaging approaches were also able to depict changes in response to the treatment. However, a continuous increase in the accumulation of Annexin Vivo was observed over time by FMT/*µ*CT imaging, whereas immunohistochemical analyses revealed a strong induction of tumor apoptosis already at day 4 after therapy start with no further increase thereafter. Thus, the early strong induction of apoptosis was not reliably captured by Annexin V-based *in vivo* apoptosis imaging. Measurement of the glucose metabolism by ^18^F-FDG-PET/CT, on the other hand, was more reliable and sensitive for the assessment of early anti-angiogenic therapy effects. This was obvious by the markedly stronger decline in the tumor-to-muscle ratio between days 0 and 4 than between days 4 and 9. The high sensitivity of ^18^F-FDG-PET for the detection of early sunitinib-induced treatment effects is in line with our previous results obtained in the A431 skin cancer model [27]. In this study, the decrease in intratumoral ^18^F-FDG uptake was highly significant even at day one after therapy start.

The lower reliability of Annexin V-based imaging to depict the early induction of tumor apoptosis in A549 tumors in response to sunitinib can be explained by an impaired delivery of the Annexin V-based imaging probe due to the strong reduction in tumor vascularization. Micro vessel density was 3.6-fold lower in sunitinib-treated A549 versus control tumors at day 4 after therapy start. Strong vessel pruning by anti-angiogenic therapy is expected to predominantly hamper the delivery of Annexin Vivo as this imaging probe has a substantially higher molecular weight than ^18^F-FDG. However, a better tumor accumulation of the Annexin V-based imaging probe was observed in the A549 lung tumors during sunitinib treatment as compared to the results that we had obtained in the highly angiogenic A431 skin cancer model [21]. This may be due to the higher fraction of mature vessels in A549 tumors (over 70%, [22] as compared to A431 with 14%, [21]), which are less prone to anti-angiogenic therapy [33] and were not significantly reduced by sunitinib. Mature vessels are generally more functional and better perfused [34]. Thus, we assume that the higher proportion of mature vessels in A549 tumors allowed a better delivery of the imaging probe to the tumor tissue. This is further supported by the observation that Annexin V-based apoptosis imaging was more reliable for the detection of the response to chemotherapy with carboplatin that had no effect on the mature vessels in A549 tumors.

Nevertheless, our findings contradict results of a previous preclinical study in which a positive correlation was found between the tumor accumulation of Annexin Vivo *in vivo* and immunohistochemical data on tumor apoptosis in response to anti-angiogenic therapy [15]. One possible explanation for the discrepancy might be that both studies were done in different tumor models. Another reason might be that, in the study performed by Kazmierczak and colleagues, the tumor vascularization was less strongly affected by the anti-angiogenic therapy. The authors used another anti-angiogenic drug (regorafenib) in a moderate dosage (10 mg/kg body weight) and observed a markedly lower reduction in tumor micro vessel density (1.7-fold versus 3.6-fold in our study). We assume that, due to these minor effects of the anti-angiogenic drug on the tumor vasculature in the study performed by Kazmierczak et al., the imaging probe had reached the tumor tissue in sufficient amounts to detect apoptosis. In a review and meta-analysis of 17 clinical trials, Belhocine and colleagues reported that the positive predictive value of Annexin V-based apoptosis imaging was high, showing the potential of Annexin V-based apoptosis imaging for predicting a positive response to therapies in case of a significant tumor accumulation of the imaging probe. However, the negative predictive value was only moderate, showing limitations of Annexin V-based imaging in predicting a negative tumor response to treatment in patients in case of a low tumor accumulation of the probe. The authors conclude that the reliability of Annexin V-based imaging is depending on the cancer type and the type of treatment, without investigating this issue any further [30]. Their observation that a negative imaging outcome does not consequently predict a negative response to the therapy is in line with the results obtained in our study and further sustains our hypothesis that the reliability of Annexin V-based apoptosis imaging can be impaired if the therapy significantly affects the tumor vasculature, leading to a reduced delivery of the probe to the tumor. For further investigations, it would be interesting to include small molecule imaging probes that detect apoptosis, e.g., analogues of duramycin [35, 36], and to compare apoptosis imaging using small molecule and Annexin V-based probes with glucose metabolism measurements for monitoring the response to different anti-cancer therapies.

## 5. Conclusion

Annexin V-based apoptosis imaging and ^18^F-FDG-based measurement of the glucose metabolism enable the assessment of cytotoxic and anti-angiogenic treatment effects in A549 tumors. However, for anti-angiogenic therapy, glucose metabolism imaging was more sensitive and detected early therapy effects more reliably. The lower sensitivity and reliability of Annexin V-based apoptosis imaging during anti-angiogenic treatment can be explained by the strong reduction in tumor vessel density and an impaired delivery of the Annexin V imaging probe to the tumor.

## Data Availability

The data used to support the findings of the study are available from the corresponding author upon request.

## Abbreviations

CT: Computed tomography
^18^F-FDG: ^18^F-fluorodeoxyglucose
FMT: Fluorescence molecular tomography
PET: Positron-emission-tomography
PS: Phosphatidylserine
SMA: Alpha smooth muscle actin
TUNEL: Terminal deoxynucleotidyl transferase dUTP Nick end labeling.

## Supplementary Materials

Supplementary Materials Supplementary Figure S1. Amount of mature vessels in tumors. Quantitative immunohistochemistry shows that carboplatin treatment has no obvious effect on the amount of mature vessels (carboplatin d4: 61.48 ± 10.25; d9: 54.38 ± 17.22 SMA-positive vessel count) (Figure S1A). In contrast, reducing numbers of mature vessels are found in response to anti-angiogenic treatment with sunitinib despite the absence of statistical significance (control: 55.57 ± 19.87; sunitinib d4: 41.33 ± 24.93; d9: 34.70 ± 21.25 SMA-positive vessel count) (Figure S1B).
