# Supplementary material for: Change of Apoptosis and Glucose Metabolism in Lung Cancer Xenografts during Cytotoxic and Anti-Angiogenic Therapy Assessed by Annexin V Based Optical Imaging and 18F-FDG-PET/CT
Source: Contrast Media Mol Imaging. 2021 Apr 10;2021:6676337. doi: 10.1155/2021/6676337 (PMC8057888; doi:10.1155/2021/6676337)
Supplement: Supplementary Materials — Supplementary Figure S1. Amount of mature vessels in tumors. Quantitative immunohistochemistry shows that carboplatin treatment has no obvious effect on the amount of mature vessels (carboplatin d4: 61.48 ± 10.25; d9: 54.38 ± 17.22 SMA-positive vessel count) (Figure S1A). In contrast, reducing numbers of mature vessels are found in response to anti-angiogenic treatment with sunitinib despite the absence of statistical significance (control: 55.57 ± 19.87; sunitinib d4: 41.33 ± 24.93; d9: 34.70 ± 21.25 SMA-positive vessel count) (Figure S1B). [file 6676337.f1.docx]

Supplementary materials


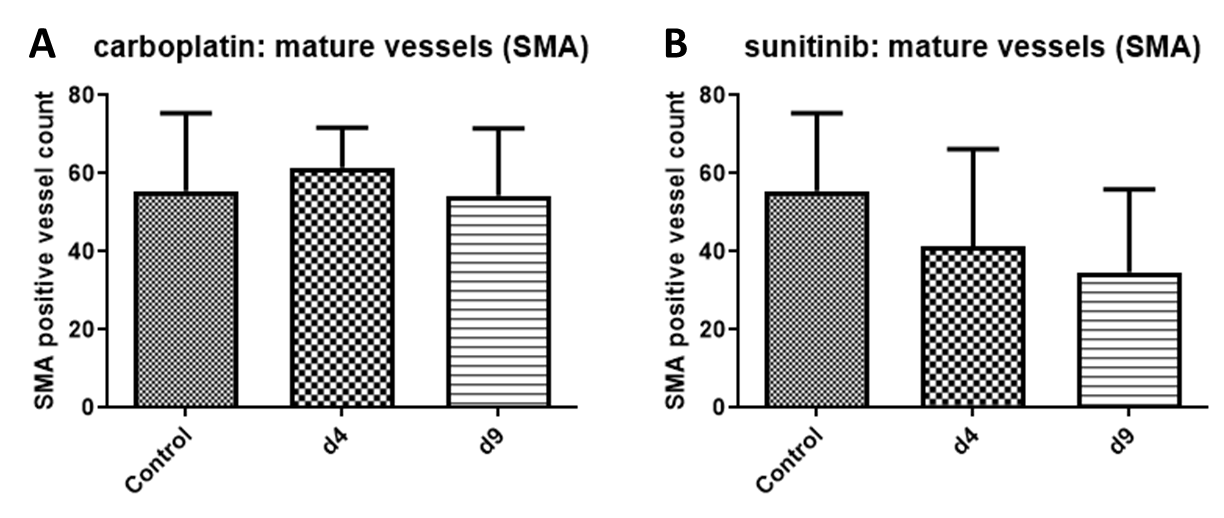


**Supplementary Figure S1: Amount of mature vessels in tumors.** Quantitative immunohistochemistry shows that carboplatin treatment has no obvious effect on the amount of mature vessels (carboplatin d4: 61.48 ± 10.25; d9 54.38 ± 17.22 SMA positive vessel count) (**Figure S1A**). In contrast, reducing numbers of mature vessels are found in response to anti-angiogenic treatment with sunitinib despite the absence of statistical significance (control: 55.57 ± 19.87; sunitinib d4: 41.33 ± 24.93; d9: 34.70 ± 21.25 SMA positive vessel count). (**Figure S1B**).
